# The Mechanism of Bronchophony

**Published:** 1852-07

**Authors:** W. H. Walshe

**Affiliations:** Professor of the Principles and Practice of Medicine, University College, London, &c.


*The Mechanism of Bronchophony.* By W. H. Walshe, M.D., Professor of the Principles and Practice of Medicine, University College, London, &c.

§ I. The physical conditions in which morbid bronchophony is observable are:

1. Increased density of the pulmonary tissue either surrounding pervious bronchi, or forming a medium of communication between pervious bronchi and the spot at the thoracic surface examined,—whether that increase of density be caused by solid, semi-solid, or, perhaps, even liquid infiltration of the parenchyma.
2. Increased density of texture, produced by extraneous pressure.
3. Presence of any solid extra-pulmonary formation in such a situation as to form a connecting link between the surface examined and a bronchus of some calibre.
4. Increased width and hypertrophy of the substance of the bronchial tubes.
5. Diminished density of the lung, as in the rarefaction of vesicular emphysema.

The diseases referable to the first head are the *common* causes of brochophony; but any hypothesis in explanation of the phenomenon must also apply to the other and comparatively *rare* causes.

§ II. Laennec regarded bronchophony as an essential dependence on increased density of the pulmonary texture, and supposed that it was simply produced by the greater facility with which comparatively dense homogeneous tissue—homogeneous from the exclusion of air—conducted the laryngeal vibrations to the surface.

This explanation is inadequate to meet all the circumstances of the case. In the first place bronchophony *may* exist, and this to an intense degree, over lung rarefied to such a degree as to give actually almost tympanitic resonance under percussion. Of this fact, (not generally known, or, at least, taught,) I have observed a certain number of examples, where *post-mortem* examination left no doubt of the absence of any textural change in the lung except emphysema. In the second place, as was, I believe, first mentioned in print by Skoda, the voice resounds sometimes with greater intensity, when ausculted over the chest than over the larynx itself. In the third place the pitch of the bronchophonic differs sometimes distinctly from that of the laryngeal voice. These facts, without disproving the correctness of Laennec's theory as far as it goes, show at least that it requires an addition of some kind to make it include all the varieties of the phenomenon.

§ III. Skoda, holding the general doctrine that the "varying conducting power of the healthy and diseased lung-substance cannot be taken as a basis of explanation of any of the phenomena of auscultation," specially opposes Laennec's views of the mechanism of bronchophony on the following grounds:

(*a*) Bronchophony may in the course of a few minutes appear and disappear over hepatised lung, the other physical signs, especially the percussion sound, having undergone no change. (*b*) When vocal resonance thus suddenly disappears, it may as suddenly be restored by making the patient cough or breathe deeply, so as to free the bronchi from fluid in the part of the organ ausculted. (*c*) In cases of pleuritic effusion, the resonance grows weaker and weaker in proportion as the fluid increases; now, as the lung grows more and more solid, the greater the quantity of pleural fluid compressing it, the reverse ought to be observed, were Laennec's doctrine of solidification and improved conducting capability well founded. (*d*) If a healthy and hepatised lung be removed from the body, and if, while one person speaks through a stethoscope placed in contact with the surface of each organ successively, a second listens through another stethoscope placed at an opposite point of that surface, the listener will find that more intense resonance reaches the ear through the healthy than the diseased lung.

What is the force of these objections? Let us examine them *seriatim.* (*a* and *b*) Since I first became acquainted with Skoda's statement concerning the appearance and disappearance of bronchophony in hepatisation, I have repeatedly endeavored to produce artificially the change he describes, but have invariably failed; I have questioned other physicians on the point, and find that they have not observed the alleged variableness in the vocal resonance. I have, however, in one or two instances, noticed intermittent bronchophony occurring spontaneously without the patient having expectorated or even coughed,—a peculiarity evidently of different mechanism from that insisted upon by Skoda, and one to which I will by-and-by return. (*c*) The argument derived from the phenomena of pleuritic effusion, seems feeble, and, indeed, unsound. The interposition of a mass of fluid between the condensed lung and the surface alters the terms of the problem completely. It has been shown by Colladon and Sturm, that sonorous rays which reach the surface of water at a very acute angle do not pass into the air, but undergo reflection in the interior of the liquid. Now the angle at which the sonorous vibrations reach the fluid from the bronchi, and ultimately reach the outer surface of the pleural fluid, may very possibly often prove of the degree of acuteness fitted to prevent their passage into the air. At all events Skoda, by ignoring the new influences likely to be exercised by the fluid, renders his argument valueless.* (*d*) The results I have obtained from some experiments on the conducting powers of hepatised tissue, do not agree with those announced by Skoda. First: It is, I admit, quite true, that tissue, called hepatised, may conduct the voice no better, or even less forcibly, than a similar thickness of healthy parenchyma; but it is equally true, that this is not a constant result. I have occasionally found hepatised lungs taken from the body, conduct the sound with extreme intensity. And these varying results may be obtained from different lungs, which the naked eye would judge to be in the *same* state physically, in regard of their shares of air, fluid, and semi-plastic substance: but it is evident, from

* Let it not be forgotten, too, that in the interscapular region strong bronchophony is sometimes audible, even where fluid is accumulated in large quantity in the pleural sac.

the variations in result, that, acoustically, they are in *different* physical states; and that, therefore, such experiments as Skoda's are not to be trusted to. Specimens of parenchyma, apparently identical, are in reality widely different. There can, for example, be little doubt, that varying homogeneousness plays a more important part, than any observable so-called solidification, in regulating the conducting power of the lung. In the varying homogeneousness of different specimens may, in truth, lie the key to the difficulty; but even, if so, it is a key which cannot practically be used. Secondly: If, while one person speaks into a stethoscope, with its narrow end introduced into the trachea, a second listens over a part of the chest where hepatised lung lies beneath, (and where intense sniffling bronchopony existed during life), the listener will often be surprised at the singular and total absence of sound. Skoda, obliged to admit this fact, attempts to evade its force by supposing the vibrations to be interfered with by fluid in the bronchi. To this, I would reply, that I have satisfied myself of the total absence of such *post-mortem* resonance over pneumonic solidification, in a case where the bronchi, to the third and fourth divisions, were peculiarly free from fluid, and scarcely any spumous liquid infiltrated the parenchyma—which very same parenchyma, removed from the body, conducted the voice from one stethoscope through another with striking intensity. If we consider the main difference in the physical conditions of the parts, when an individual himself speaks, or when another speaks into his trachea after his death, an obvious explanation of the experimental failures to imitate the bronchophony of life suggests itself. In the dead body, in truth, the laryngeal, tracheal, and bronchial walls take no part in the production or conduction of the sound, which is propagated by their contained air alone; whereas, in life, the walls of those tubes obviously conduct the sonorous vibrations. But to admit this, would have been fatal to more than one of Skoda's hypotheses. Besides, hepatised and healthy lungs are not strictly comparable in and out of the body in regard of this matter; *within the body* the contact of a hepatised lung with the chest-wall is more perfect than that of a healthy one; and, admitting that the former is a worse conductor in regard of the condition of its substance, it may be a much better one through the closeness of its union (especially if adhesive) with the parietes. Here is a point which has been totally overlooked by the Viennese physician and his followers.

§ IV. I conceive, then, that, whether Laennec's doctrine be true or false, the arguments just reviewed fail to prove it unsound. Skoda himself naturally thinks otherwise; and, excluding the walls of the trachea and bronchi from all share in the *conduction* of the sonorous vibrations of the chordæ vocales,* (an office assigned by him to the contained air of those tubes alone,) maintains that bronchophony is really pro-

* Skoda, of course, admits, that the bronchial walls, in the spot where consonance takes place, intensify the consonating tones of the air within them by their own vibration; but this is a very different thing from their taking part in the production of bronchophony by conducting the laryngeal voice.

duced by the consonance of the air in the bronchial tubes with the laryngeal voice.

The hypothesis of consonance does not appear to me satisfactory; and it unquestionably fails to meet all the conditions of the problem. The reasons on which I ground this opinion are as follows :—(*a*) Air in any enclosed space does not consonate with every sound produced at its orifice, but only with the fundamental note of that space, and with certain others having a fixed harmonical relationship to that fundamental note,—with certain of its *concords*, in short. This is easily ascertained, in a rough way, by running the gamut with the voice at the mouth of an empty water-bottle; one note only of the octave is at all markedly reinforced by consonance within the cavity,—one or two others, (according to the distance from the orifice at which the vocal sound is emitted, and the depth of the mass of air within the bottle,) very slightly increased in loudness. Now, on the contrary, when bronchophony exists, it is audible with *successive notes* of the octave, standing in no harmonical relationship to each other—absolute *discords*, in short. These successive notes are to be found at the lowest part of each register, (whether bass, tenor, or soprano); but the force of bronchophony gradually decreases, not at harmonic intervals, but on *each successive note from below upwards*, until the resonance disappears altogether;* and obviously the limitation of bronchophony to grave tones, as contra-distinguished to acute ones, has nothing to do with the principle of consonance, but depends upon the difference in the number of vibrations and breadth of the sonorous wave, when notes of these two classes are severally generated. (*b*) Bodies consonate only in unison, or in certain fixed harmony, with the original sound which throws them into vibration.† Now, the pitch of the bronchophonic voice varies irregularly from that of the laryngeal with which it co-exists. This difference of pitch is especially to be caught in cases of hepatisation, and is sometimes very striking in amount; the corresponding notes heard in the larynx and on the surfaces of the chest are then, very perceptibly, discords. (*c*) Skoda's exclusion of the tracheal and bronchial walls from all participation in the transmission of the laryngeal voice is at variance both with theory and experiment, and cannot for a moment be acceded to. (*d*) In cases where the bronchophonic voice is very positively and notably louder than the laryngeal, it is difficult to believe, from the mere fact of the intensity of the sound, that the phenomenon can be due to consonance. For a consonating sound, as a rule, is vastly more feeble than the primitive tone eliciting it; and the nicest adjustment of the quantity of air in the consonating body (presuming this to be hollow) is required, in order to produce any serious increase in the amount of intensity. Let it, however, be granted, *argumenti gratia*, that chance

*Curiously enough, the vocal *resonance* always ceases to be perceptible at a lower point of the scale, than the vocal *fremitus*: this is another difference to add to numerous others distinguishing the properties of the two phenomena.

†The unison-note alone is distinct to ordinary ears; the consonating harmonies are so faint, as to require the organ of a Costa for their detection.

may sometimes cause the column of air between the larynx and the seat of bronchophony to be of the appropriate length to produce a marked increase of sound—the doctrine of Skoda gains nothing by the concession. For, be it remembered, Skoda rejects conduction as an element of bronchophony—bronchophonic voice is, in his apprehension, consonating voice; consonating voice is, then, under the above circumstances, by admission, louder than the original voice. Now, here is an idea irreconcilable with observation; for, it does not appear, that (provided the original and consonating sounds be produced by bodies of the same class, as vibrating strings, hollow bodies, solid plates, etc.) the consonating sound is ever louder than the original tone.* (*e*) If the excess of loudness of the bronchophonic over the laryngeal voice were from consonance, *vocal fremitus* (inasmuch as the walls of the consonating tubes must vibrate in the direct ratio of the vibration of their contained air) ought to rise and fall exactly as *vocal resonance.* Now, in point of fact, these two phenomena do not invariably maintain any direct relationship to each other; one may gain, while the other loses, in intensity.

These objections appear to me conclusive against the pure doctrine of consonance, as taught by Skoda, while they show that, if consonance plays any part in the production of bronchophony, it must be a subsidiary, rare, and accidental one. And even this concession is rather made on the ground, that the occurrence of consonance within the chest is not, *a priori*, impossible, than in deference to the arguments actually adduced in its favor.

§ V. From the discussion into which I have now entered, it would follow that the mechanism of bronchophony is probably complex, and certainly not, as the attempt is commonly made to prove it, invariably one and the same.

There are four points which appear especially worthy of consideration: the conduction of laryngeal voice; its possible increase of intensity within the chest; the distance at which that increase of intensity (if real) occurs from the part of the thoracic surface examined; and the relationship of pitch of the laryngeal and bronchophonic voices.

1st. In regard of conduction of laryngeal sound, theory would support the inference, that as the human voice is best propagated in air, the more the lungs were rarified, the higher would their conducting power become; and, in accordance with this, it is certain that intense bronchophony is sometimes heard over highly emphysematous tissue. However, on the other hand, as the tracheal and bronchial walls themselves vibrate

* The case may be very different where vibrations are communicated from a body of one physical constitution to a consonating body of another class. Thus, where a tube takes up the vibrations of a solid disc, the consonating note of the tube may (by managing carefully the length of the vibrating column of air in the interior) be made incomparably more powerful than the original tone; the quality of such notes is exquisitely pure and full; and, long ago, Savart suggested the construction of a musical instrument on this principle, which, it seemed probable, would exceed in melody and power any of those in use. Civilised musicians have not profited by the philosopher's suggestion; yet the savages of some of the Pacific islands, curiously enough, have hit upon rude contrivances illustrating the principle.

during speaking, any really solid material directly connecting a large bronchus with the surface of the chest, must conduct those vibrations forcibly; and, in accordance with this, we find that wherever solid fibrous structure is seated in the manner supposed, bronchophony of the most intense character is audible. But if the union of the solid material with the chest-wall be imperfect, if there be any interruption at the planes of union of the conducting materials, the acoustic conditions are completely changed, inasmuch as interruption at the union of media of different densities most deeply impairs the conducting faculty of the series. Here is one clue to the differences in vocal resonance, observed in cases where the physical conditions appear, on superficial view, identical; hence, too, we have no fair reason to expect that, in all samples of the variable semi-solid states comprised under the title of "hepatisation," conducting power should be affected in an uniform manner, and experiment shows that it actually is not. Experiment, in truth, alone can teach, in each instance, what the force of conduction really is in the various complicated conditions of physical change in the lungs.

2ndly. It is indubitable, that the bronchophonic voice is sometimes louder than that transmitted through the stethoscope directly from the larynx. The extreme rarity of this occurrence does not affect its reality; and hence, some explanation must be found for the increase of intensity within the thorax. We have seen the objections to Skoda's hypothesis of consonance. Now, there is another way in which the intensity of sounds may be augmented at a greater or less distance from their place of production—by reflection, and by reflections brought to a focus, or *echo.* But are the conditions of reflection fulfilled in the hepatised lung? It would appear so; for the tubes along which the voice is transmitted from the larynx are surrounded by semi-solid material, proper (when compared with healthy tissue) to reflect and concentrate the sound; while the air-cells and minute bronchi are closed to a variable distance, and prevent its divergence. The tubes resemble so many speaking-trumpets, and, just as in these instruments, the augmentation of sound is produced by reflection from their quivering walls; as this reflection tends to propagate vibrations (otherwise divergent) in the same direction, increased intensity of sound must be the result. And, further, if the reflected vibrations chance to be brought to a focus within a large tube, then *echo* will occur; and, as under ordinary circumstances, the echo may be materially louder than the original sound. But, it may be inquired, how, upon this theory, is the temporary disappearance of bronchophony, already referred to as a possible, though very rare occurrence, explicable? It is conceivable, that where such suspension occurs, this may sometimes depend on the deadening influence of accumulated fluid in the tubes.* It may also be sometimes traced to accidental pressure of a main tube by some extraneous body, which, from movements of the trunk, the act of coughing, &c., presses much or little, or not at all,

* The effect of carpeting or woollen cloth of any kind, in deadening the sound of music in an apartment is well known. The intermixture of air and solid fibres in the carpets, through which the sound has to pass, deadens the echo upon that tube.* It seems extremely probable that, under the latter circumstances, bronchophony would, (I have not, however, verified this conjecture,) be heard at some point of the chest nearer the bifurcation of the trachea. Again, it is possible that certain changes of posture, altering the relationship of the reflecting surfaces, might interfere with the production of echo, by preventing the reflected sounds from coming to their usual focus. Besides, the position of the auscultator in respect of the focal point, might prevent him from hearing an echo really existing.† The force of the echo will also rise, the smoother the bronchial walls, and the larger the tubes in which it occurs. And numerous other circumstances may be conceived, but scarcely proved, to affect the phenomenon. Among these, the composition of the gases within the bronchi may, for aught that is known, hold an important place; hydrogen has been proved to deaden sound greatly; the effect of carbonic acid, mixed with other gases and aqueous vapor can only be ascertained from experiment.

Thirdly. As concerns the distance from the point of auscultation at which the reinforcement of sound within the thorax occurs: the further away, the less of the resonance will reach the surface; the amount, however, will be modified by the conducting property of the interposed media.

A little consideration will show that these three conditions of bronchophony—conduction of laryngeal sound, increased intensity of this within the thorax, and proximity of site of the increase—may or may not be directly as each other; one may be in a state favorable to, the rest unfavorable to, the formation of bronchophony. Hence the variable state of the sign in different cases of the same disease; and hence an additional clue, for example, to the inconstancy of bronchophony in pleuritic effusion.

Fourthly. The relationship of pitch of the bronchophonic and laryngeal voices seems the most difficult part of the subject—difficult, at least, in those cases where a distinct difference can be detected, in the pitch of the two. The consideration of this point is reserved for another occasion.—*London Med. Times.*

between the ceiling and floor, by which the original sound is swelled."—Herschel, art, "Sound," *Encyc. Metrop.* Aerated mucus and sanguineous serum in the bronchi would have the same effect on vocal echo in those tubes, as the carpeting under the circumstances referred to above.

* This cause is suggested from actual observation.

†The existing theory of echoes generally is inadequate to explain many of their phenomena. There is (or was) a ruined fortress near Louvain well illustrating this. Here, if a person sings, he only hears his own voice, without any repetition; those who stand at some distance hear the echo, but not the voice,—and they hear the echo with surprising variations,—sometimes louder, sometimes softer,—now near, now distant.—*Burrowes's Cyclop.*, *Art.* "*Acoustics.*"

